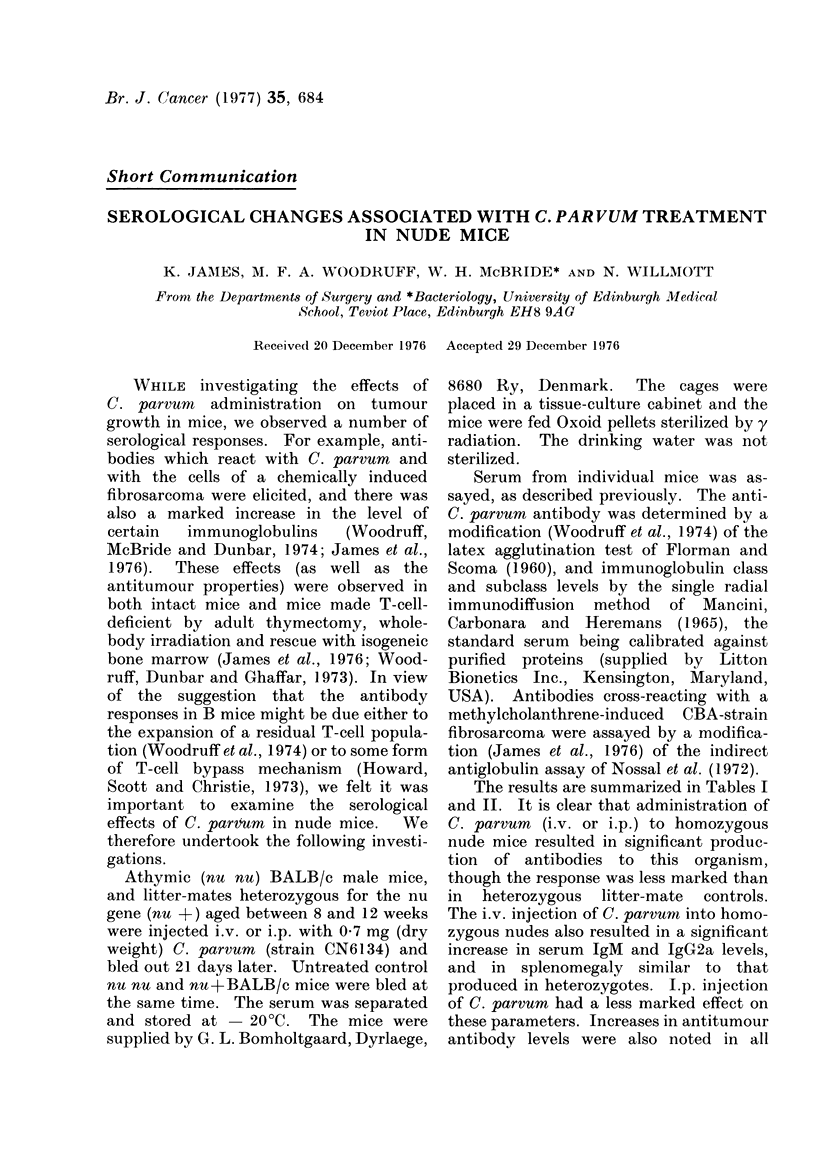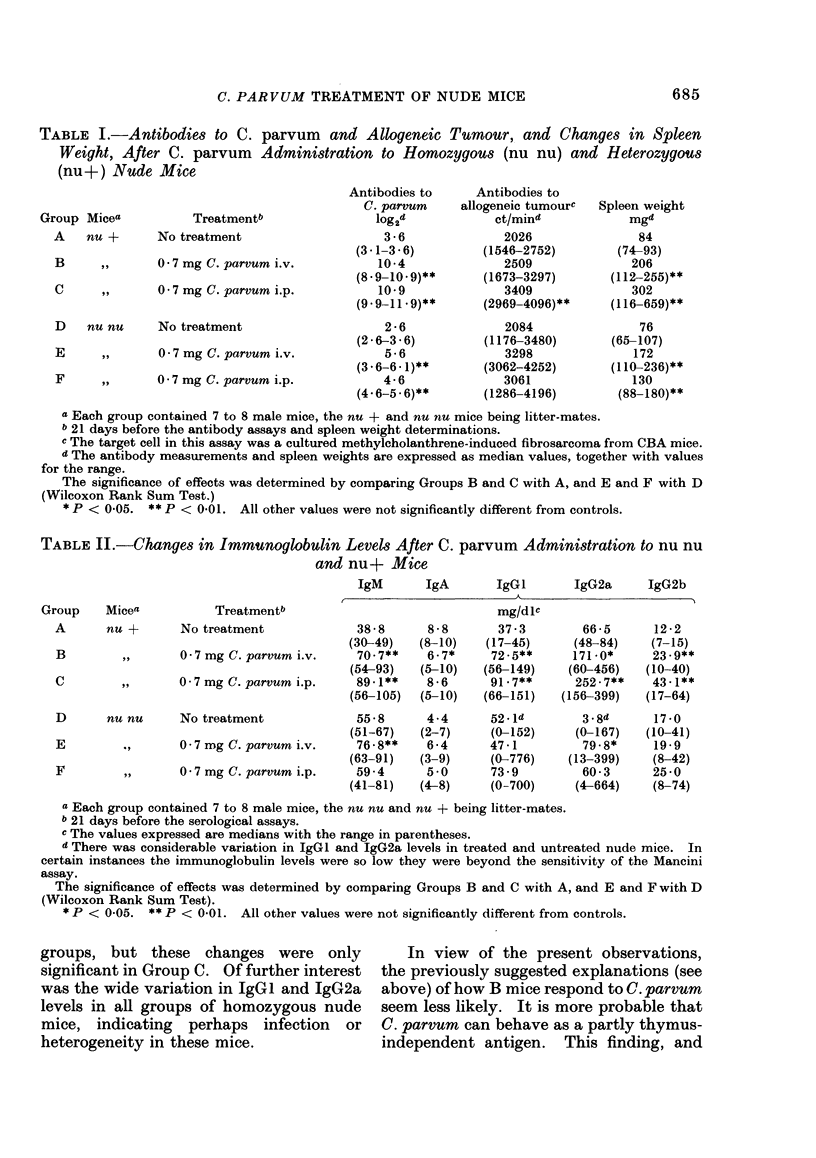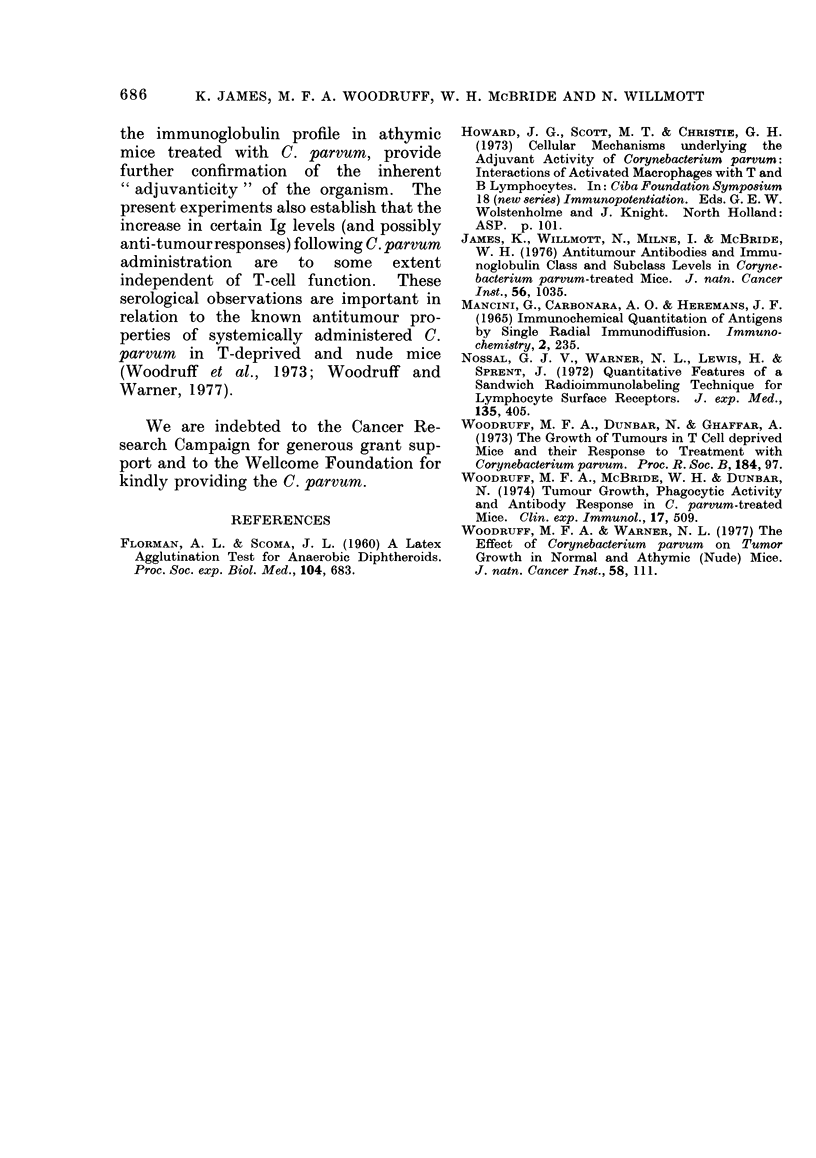# Serological changes associated with C. parvum treatment in nude mice.

**DOI:** 10.1038/bjc.1977.104

**Published:** 1977-05

**Authors:** K. James, M. F. Woodruff, W. H. McBride, N. Willmott


*Br. J. Cancer* (1977) **35**, 684

### *Short Communication*

### SEROLOGICAL CHANGES ASSOCIATED WITH *C. PARVUM* TREATMENT IN NUDE MICE

K. JAMES, M. F. A. WOODRUFF, W. H. McBRIDE* AND N. WILLMOTT

*From the Departments of Surgery and *Bacteriology, University of Edinburgh Medical School, Teviot Place, Edinburgh EH8 9AG*

Received 20 December 1976  Accepted 29 December 1976

WHILE investigating the effects of *C. parvum* administration on tumour growth in mice, we observed a number of serological responses. For example, antibodies which react with *C. parvum* and with the cells of a chemically induced fibrosarcoma were elicited, and there was also a marked increase in the level of certain immunoglobulins (Woodruff, McBride and Dunbar, 1974; James *et al.*, 1976). These effects (as well as the antitumour properties) were observed in both intact mice and mice made T-cell-deficient by adult thymectomy, whole-body irradiation and rescue with isogeneic bone marrow (James *et al.*, 1976; Woodruff, Dunbar and Ghaffar, 1973). In view of the suggestion that the antibody responses in B mice might be due either to the expansion of a residual T-cell population (Woodruff *et al.*, 1974) or to some form of T-cell bypass mechanism (Howard, Scott and Christie, 1973), we felt it was important to examine the serological effects of *C. parvum* in nude mice. We therefore undertook the following investigations.

Athymic (*nu nu*) BALB/c male mice, and litter-mates heterozygous for the nu gene (*nu* +) aged between 8 and 12 weeks were injected i.v. or i.p. with 0·7 mg (dry weight) *C. parvum* (strain CN6134) and bled out 21 days later. Untreated control *nu nu* and *nu*+BALB/c mice were bled at the same time. The serum was separated and stored at − 20°C. The mice were supplied by G. L. Bomholtgaard, Dyrlaege, 8680 Ry, Denmark. The cages were placed in a tissue-culture cabinet and the mice were fed Oxoid pellets sterilized by $\gamma$ radiation. The drinking water was not sterilized.

Serum from individual mice was assayed, as described previously. The anti-*C. parvum* antibody was determined by a modification (Woodruff *et al.*, 1974) of the latex agglutination test of Florman and Scoma (1960), and immunoglobulin class and subclass levels by the single radial immunodiffusion method of Mancini, Carbonara and Heremans (1965), the standard serum being calibrated against purified proteins (supplied by Litton Bionetics Inc., Kensington, Maryland, USA). Antibodies cross-reacting with a methylcholanthrene-induced CBA-strain fibrosarcoma were assayed by a modification (James *et al.*, 1976) of the indirect antiglobulin assay of Nossal *et al.* (1972).

The results are summarized in Tables I and II. It is clear that administration of *C. parvum* (i.v. or i.p.) to homozygous nude mice resulted in significant production of antibodies to this organism, though the response was less marked than in heterozygous litter-mate controls. The i.v. injection of *C. parvum* into homozygous nudes also resulted in a significant increase in serum IgM and IgG2a levels, and in splenomegaly similar to that produced in heterozygotes. I.p. injection of *C. parvum* had a less marked effect on these parameters. Increases in antitumour antibody levels were also noted in all

TABLE I.—*Antibodies to* C. parvum *and Allogeneic Tumour, and Changes in Spleen Weight, After* C. parvum *Administration to Homozygous* (nu nu) *and Heterozygous* (nu+) *Nude Mice*

| Group | Mice[a] | Treatment[b] | Antibodies to *C. parvum* $\log_2$[d] | Antibodies to allogeneic tumour[c] ct/min[d] | Spleen weight mg[d] |
|---|---|---|---|---|---|
| A | *nu* + | No treatment | 3·6 (3·1–3·6) | 2026 (1546–2752) | 84 (74–93) |
| B | ,, | 0·7 mg *C. parvum* i.v. | 10·4 (8·9–10·9)** | 2509 (1673–3297) | 206 (112–255)** |
| C | ,, | 0·7 mg *C. parvum* i.p. | 10·9 (9·9–11·9)** | 3409 (2969–4096)** | 302 (116–659)** |
| D | *nu nu* | No treatment | 2·6 (2·6–3·6) | 2084 (1176–3480) | 76 (65–107) |
| E | ,, | 0·7 mg *C. parvum* i.v. | 5·6 (3·6–6·1)** | 3298 (3062–4252) | 172 (110–236)** |
| F | ,, | 0·7 mg *C. parvum* i.p. | 4·6 (4·6–5·6)** | 3061 (1286–4196) | 130 (88–180)** |

[a] Each group contained 7 to 8 male mice, the *nu* + and *nu nu* mice being litter-mates.
[b] 21 days before the antibody assays and spleen weight determinations.
[c] The target cell in this assay was a cultured methylcholanthrene-induced fibrosarcoma from CBA mice.
[d] The antibody measurements and spleen weights are expressed as median values, together with values for the range.
The significance of effects was determined by comparing Groups B and C with A, and E and F with D (Wilcoxon Rank Sum Test.)
* $P < 0{\cdot}05$. ** $P < 0{\cdot}01$. All other values were not significantly different from controls.

TABLE II.—*Changes in Immunoglobulin Levels After* C. parvum *Administration to* nu nu *and* nu+ *Mice*

| Group | Mice[a] | Treatment[b] | IgM | IgA | IgG1 | IgG2a | IgG2b |
|---|---|---|---|---|---|---|---|
| | | | mg/dl[c] | | | | |
| A | *nu* + | No treatment | 38·8 (30–49) | 8·8 (8–10) | 37·3 (17–45) | 66·5 (48–84) | 12·2 (7–15) |
| B | ,, | 0·7 mg *C. parvum* i.v. | 70·7** (54–93) | 6·7* (5–10) | 72·5** (56–149) | 171·0* (60–456) | 23·9** (10–40) |
| C | ,, | 0·7 mg *C. parvum* i.p. | 89·1** (56–105) | 8·6 (5–10) | 91·7** (66–151) | 252·7** (156–399) | 43·1** (17–64) |
| D | *nu nu* | No treatment | 55·8 (51–67) | 4·4 (2–7) | 52·1[d] (0–152) | 3·8[d] (0–167) | 17·0 (10–41) |
| E | ,, | 0·7 mg *C. parvum* i.v. | 76·8** (63–91) | 6·4 (3–9) | 47·1 (0–776) | 79·8* (13–399) | 19·9 (8–42) |
| F | ,, | 0·7 mg *C. parvum* i.p. | 59·4 (41–81) | 5·0 (4–8) | 73·9 (0–700) | 60·3 (4–664) | 25·0 (8–74) |

[a] Each group contained 7 to 8 male mice, the *nu nu* and *nu* + being litter-mates.
[b] 21 days before the serological assays.
[c] The values expressed are medians with the range in parentheses.
[d] There was considerable variation in IgG1 and IgG2a levels in treated and untreated nude mice. In certain instances the immunoglobulin levels were so low they were beyond the sensitivity of the Mancini assay.
The significance of effects was determined by comparing Groups B and C with A, and E and F with D (Wilcoxon Rank Sum Test).
* $P < 0{\cdot}05$. ** $P < 0{\cdot}01$. All other values were not significantly different from controls.

groups, but these changes were only significant in Group C. Of further interest was the wide variation in IgG1 and IgG2a levels in all groups of homozygous nude mice, indicating perhaps infection or heterogeneity in these mice.

In view of the present observations, the previously suggested explanations (see above) of how B mice respond to *C. parvum* seem less likely. It is more probable that *C. parvum* can behave as a partly thymus-independent antigen. This finding, and the immunoglobulin profile in athymic mice treated with *C. parvum*, provide further confirmation of the inherent "adjuvanticity" of the organism. The present experiments also establish that the increase in certain Ig levels (and possibly anti-tumour responses) following *C. parvum* administration are to some extent independent of T-cell function. These serological observations are important in relation to the known antitumour properties of systemically administered *C. parvum* in T-deprived and nude mice (Woodruff *et al.*, 1973; Woodruff and Warner, 1977).

We are indebted to the Cancer Research Campaign for generous grant support and to the Wellcome Foundation for kindly providing the *C. parvum*.

### REFERENCES

FLORMAN, A. L. & SCOMA, J. L. (1960) A Latex Agglutination Test for Anaerobic Diphtheroids. *Proc. Soc. exp. Biol. Med.*, **104**, 683.

HOWARD, J. G., SCOTT, M. T. & CHRISTIE, G. H. (1973) Cellular Mechanisms underlying the Adjuvant Activity of *Corynebacterium parvum*: Interactions of Activated Macrophages with T and B Lymphocytes. In: *Ciba Foundation Symposium* 18 (*new series*) *Immunopotentiation*. Eds. G. E. W. Wolstenholme and J. Knight. North Holland: ASP. p. 101.

JAMES, K., WILLMOTT, N., MILNE, I. & McBRIDE, W. H. (1976) Antitumour Antibodies and Immunoglobulin Class and Subclass Levels in *Corynebacterium parvum*-treated Mice. *J. natn. Cancer Inst.*, **56**, 1035.

MANCINI, G., CARBONARA, A. O. & HEREMANS, J. F. (1965) Immunochemical Quantitation of Antigens by Single Radial Immunodiffusion. *Immunochemistry*, **2**, 235.

NOSSAL, G. J. V., WARNER, N. L., LEWIS, H. & SPRENT, J. (1972) Quantitative Features of a Sandwich Radioimmunolabeling Technique for Lymphocyte Surface Receptors. *J. exp. Med.*, **135**, 405.

WOODRUFF, M. F. A., DUNBAR, N. & GHAFFAR, A. (1973) The Growth of Tumours in T Cell deprived Mice and their Response to Treatment with *Corynebacterium parvum*. *Proc. R. Soc. B*, **184**, 97.

WOODRUFF, M. F. A., McBRIDE, W. H. & DUNBAR, N. (1974) Tumour Growth, Phagocytic Activity and Antibody Response in *C. parvum*-treated Mice. *Clin. exp. Immunol.*, **17**, 509.

WOODRUFF, M. F. A. & WARNER, N. L. (1977) The Effect of *Corynebacterium parvum* on *Tumor* Growth in Normal and Athymic (Nude) Mice. *J. natn. Cancer Inst.*, **58**, 111.